# Of Amoebae and Men: Extracellular DNA Traps as an Ancient Cell-Intrinsic Defense Mechanism

**DOI:** 10.3389/fimmu.2016.00269

**Published:** 2016-07-08

**Authors:** Xuezhi Zhang, Thierry Soldati

**Affiliations:** ^1^Department of Biochemistry, Science II, University of Geneva, Geneva, Switzerland

**Keywords:** amoebozoa, Dictyostelium, NOX, neutrophil extracellular traps, evolution, unicellular eukaryotes, multicellularity, innate immunity

## Abstract

Since the discovery of the formation of DNA-based extracellular traps (ETs) by neutrophils as an innate immune defense mechanism (1), hundreds of articles describe the involvement of ETs in physiological and pathological human and animal conditions [reviewed in Ref. (2), and the previous Frontiers Research Topic on NETosis: http://www.frontiersin.org/books/NETosis_At_the_Intersection_of_Cell_Biology_Microbiology_and_Immunology/195]. Interestingly, a few reports reveal that ETs can be formed by immune cells of more ancient organisms, as far back as the common ancestor of vertebrates and invertebrates (3). Recently, we reported that the Sentinel cells of the multicellular slug of the social amoeba *Dictyostelium discoideum* also produce ETs to trap and kill slug-invading bacteria [see Box 1; and Figure 1 Ref. (4)]. This is a strong evidence that DNA-based cell-intrinsic defense mechanisms emerged much earlier than thought, about 1.3 billion years ago. Amazingly, using extrusion of DNA as a weapon to capture and kill uningestable microbes has its rationale. During the emergence of multicellularity, a primitive innate immune system developed in the form of a dedicated set of specialized phagocytic cells. This professionalization of immunity allowed the evolution of sophisticated defense mechanisms including the sacrifice of a small set of cells by a mechanism related to NETosis. This altruistic behavior likely emerged in steps, starting from the release of “dispensable” mitochondrial DNA by *D. discoideum* Sentinel cells. Grounded in this realization, one can anticipate that in the near future, many more examples of the invention and fine-tuning of ETs by early metazoan ancestors will be identified. Consequently, it can be expected that this more complete picture of the evolution of ETs will impact our views of the involvement and pathologies linked to ETs in human and animals.

During early evolution of multicellularity, when autonomous eukaryotic single-cell hosts were encountering prokaryotes, they either phagocytosed them as food or moved away to avoid being infected. However, when multicellular organisms evolved, they had to face more directly a serious problem, namely, infection of only parts or tissues of the organism. One solution is what happens in slugs of *D. discoideum*, in which invading bacteria are trapped by patrolling S cells that are subsequently shed behind during slug migration, keeping the multicellular structure free from infection (4, 8). The phagocytes in higher animals and men follow similar strategies to circumscribe the infection. For example, patrolling neutrophils catch the invaders and commit suicide, being finally discarded by the intervention of macrophages (9). However, in plants, which are immobile and do not have circulating (innate immune) cells, and whose cells have a rigid wall, another strategy had to be co-opted to isolate infected parts from healthy tissues. One such solution is the formation of a callus or tumor induced by wounding and pathogen infections (10). Naturally, a logical question is whether extracellular DNA is also involved in plant callus and tumor formation, a topic unaddressed yet, but that might reveal interesting aspects of the evolution of innate immune defenses in the broadest way.

Box 1 **Dictyostelium discoideum as a unique model to study evolution of innate immunity.**

The social amoeba *Dictyostelium discoideum* belongs to the Amoebozoa, a sister group to the animals and fungi that branched after the divergence of plants (5, 6). The life cycle of *D. discoideum* comprises two major stages, a single-celled amoeboid stage and a “social” facultative multicellular stage. During the former, amoebae feed on bacteria and yeasts by phagocytosis, a biological process extremely well conserved in evolution and essentially shared between protozoan phagocytes and phagocytes of the animal innate immune system (7). These features make this genetically tractable organism a unique model to study the function of specific genes involved in the early evolution of innate immunity and the emergence of multicellularity.

**Figure 1:**
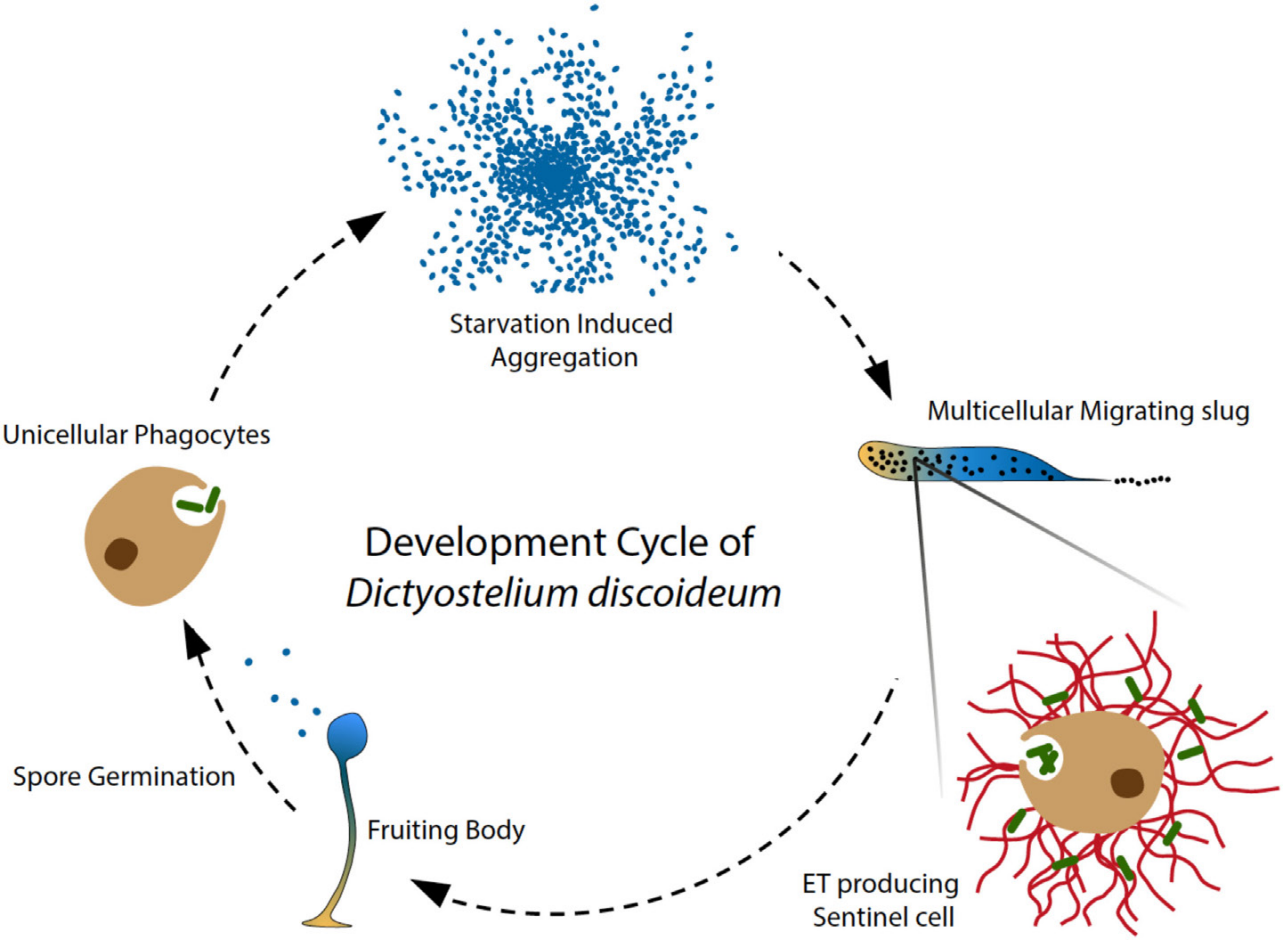
**Amoeba phagocytes and Sentinel cells capture and kill bacteria**. In the soil, solitary *D. discoideum* cells feed on bacteria, and starvation induces a developmental program, in which around 100,000 amoebae aggregate to form a migrating multicellular slug, followed by terminal differentiation and generation of fruiting bodies (28). During the migrating slug stage, only a few specialized cells, namely Sentinel (S) cells, keep their original phagocytic capacity and circulate through the slug to capture and kill invading microbes, functioning as a primitive innate immune system at the emergence of multicellular organism (8). In addition, the phagocytic S cells are constantly generated and sloughed off as the slug migrates. Our recent discovery showed that the S cells in the migrating slug of *D. discoideum* can produce extracellular DNA traps in a process that depends on production of reactive oxygen species (ROS) by NOX enzymes. Interestingly, S cells appear to mainly use their mitochondrial DNA to build up ETs, dissociating trap formation from immediate cells death by NETosis. Our study revealed that ET formation is a widespread DNA-based host defense strategy that may have been present in the ancestor of metazoa and amoebozoa.

*Dictyostelium discoideum* is a remarkable model organism to study the functions of specific genes involved in the emergence of multicellularity and the early evolution of cell autonomous defenses (8). Our recent study revealed that extracellular trap (ET) generation evolved much earlier than the emergence of metazoan and that reactive oxygen species (ROS) generated by NADPH oxidases (NOX) are essential in this conserved process (4). Therefore, in this perspective article, we would like to present the provocative hypothesis that, within some limits, the evolutionary history of ROS-generating NOX enzymes may serve as a general signature, a guiding principle that will be useful for the future discovery and study of ETs in other ancient organisms. At this point and before we develop further our arguments in favor of this causal relationship, we would like the reader to note that NADPH- or ROS-independent pathways may also contribute to ET formation under specific stimulations and conditions (11–14), an emerging field that was comprehensively reviewed by Stoiber et al. (15). Although different groups of organisms may employ different molecular machineries to fine-tune the production of ETs, and the sources of DNA may also vary depending on the process involved (1, 2, 4, 16, 17), but from a conceptual point of view, the strategies are similar, using DNA as a weapon for host defense.

Nevertheless, based on our previous phylogenetic investigations, ROS-generating NOX enzymes evolved from metal reducing ferric reductases (FRE) through a functional shift (18). As shown in Table 1, one could speculate that the organisms that express NOX homologs can generate ROS as signaling molecules to trigger ET formation for host defense. Interestingly, the evolutionary time of emergence of experimentally confirmed ET formation, NOX function, and multicellularity coincide well, possibly indicating that ROS-dependent DNA-based host defenses played a critical role in the early evolution of multicellular organisms guarded by an innate immune system.

**Table 1:** **The co-emergence of NOX enzymes and multicellularity might also correlate with the origin of DNA-based defense strategies**.

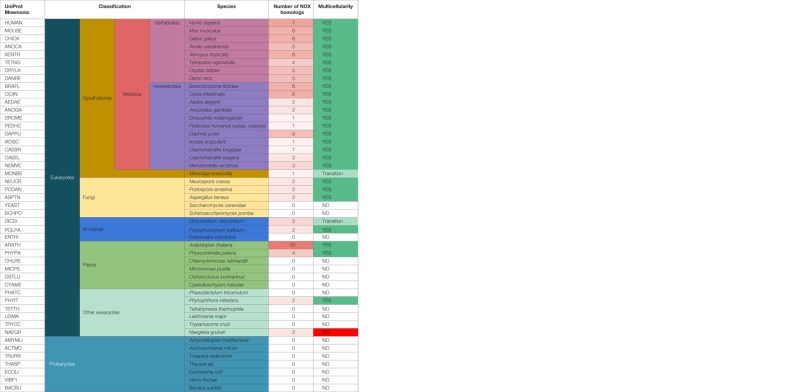

*Representative organisms from both eukaryotes and prokaryotes [see Ref. (18) for detailed presentation] were collected and organized by major branches in taxonomy. The number of NOX enzymes in each organism is indicated and color coded. Unicellular and multicellular organisms are indicated by a “NO” and “YES,” respectively. Two organisms that are at the transition between the two life forms or have both life forms are indicated as “Transition.” One exception is *Naegleria gruberi*, a single-celled organism well known for its capacity to transition from an amoeboid to a flagellated form. It is a free-living organism, but closely related to pathogenic, parasitic species. Therefore, it is plausible that the NOX gene of *Naegleria* might have been acquired from its host *via* horizontal gene transfer or that it derives from an organism that was at the transition to multicellularity, but lost this characteristic of multicellular organisms as it specialized to its environment. The discovery of NOX-dependent ET generation in the multicellular form of the amoeba *D. discoideum*, an organism that is at the transition to multicellularity, combined to the recognition of the apparent coemergence of multicellularity and NOX enzymes indicate that the origin of ET formation might be traced back to the emergence of multicellular organisms. It also suggests that variants and diverse evolutions of DNA-based defense strategies might be identified in other organisms with functional NOX enzymes, both in primitive metazoans and organisms close to the transition to multicellularity*.

In the near future, DNA-based host defense strategies will certainly be identified in a growing number of organisms. We propose that their study will reveal the fundamental significance in the relationship between host organisms and their coexisting commensals and pathogens and bring conceptual changes in the way we approach many relevant human diseases. For example, in higher plants, the roots have direct contact with various microbes in the soil, and among the various host defense mechanisms, the root border cells are able to secrete extracellular DNA to trap and kill bacteria and fungi (19, 20). In analogy, the human gut is colonized by large numbers of microbes, collectively referred to as the microbiota (21). While maintaining intimate contact with the normal microbiota, the intestinal epithelial cells are at the front of host–microbe interactions (22). Enteric pathogenic bacterial infection and antibiotic treatment are able to dramatically change the metabolic profile of the human microbiota and the gut ecosystem, sometimes leading to systemic inflammation and autoimmune responses. Importantly, unregulated ET formation is a major inducing factor of systemic inflammation and autoimmune diseases in human (23–27). Therefore, understanding whether intestinal macrophages or neutrophils, or some other intestinal cell types, are able to excrete DNA during enteric pathogenic bacterial infections or antibiotic treatment is an interesting but underexplored area. In the future, understanding the chemical dialogs between gut microbiota and ET formation could potentially lead to new therapies to control and cure these diseases.

In conclusion, formation of ETs is an ancient cell-intrinsic defense mechanism that might have played a critical role in the evolution of multicellular organisms, and we need more systematic approaches and a broader perspective to recognize the importance of ETs in host–commensal and host–pathogen interactions. We expect that more related studies in the future will keep up the excitement in this field of research.

## Author Contributions

XZ and TS designed the experiments and interpreted the results. XZ performed the experiments and XZ and TS wrote the manuscript.

## Conflict of Interest Statement

The authors declare that the research was conducted in the absence of any commercial or financial relationships that could be construed as a potential conflict of interest.
